# Gallaecimonas zhanjiangensis sp. nov., a novel member of the phylum Pseudomonadota isolated from mangrove sediments

**DOI:** 10.1099/ijsem.0.007353

**Published:** 2026-11-12

**Authors:** Min Shi, Yanyan Wang, Hanqiao Hu, Yang Jia, Baisuo Zhao, John L. Zhou, Lei Ren

**Affiliations:** 1 College of Coastal Agricultural Sciences, Guangdong Ocean University, Zhanjiang 524088, PR China; 2 College of Life and Environmental Science, Wenzhou University, Wenzhou 325035, PR China; 3 Graduate School of Chinese Academy of Agricultural Sciences, Beijing 100081, PR China; 4 Faculty of Science and Engineering, University of Nottingham Ningbo China, Ningbo 315100, PR China

**Keywords:** *Gallaecimonas*, mangrove sediment, novel species

## Abstract

A bacterial strain, designated RL-SH01^T^, was isolated from mangrove sediments in Zhanjiang, Guangdong Province, China. Strain RL-SH01^T^ is a Gram-negative, rod-shaped and flagellated bacterium. It can grow at 15–35 °C (optimum, 25–35 °C), at pH 4–10 (optimum, pH 5.0–9.0) and with salinities of 0–8% (*w/v*) (optimum, 0.5–5.0%). Phylogenetic analysis based on 16S rRNA gene sequences showed that strain RL-SH01^T^ belongs to the genus *Gallaecimonas*. Genome-wide digital DNA–DNA hybridization (dDDH) analysis showed that the dDDH values between strain RL-SH01^T^ and the other type strains of genus *Gallaecimonas* were below the 70% species delineation threshold. The major fatty acids were C_16 : 0_, Summed Feature 3 (C_16 : 1_* ω6*c and/or C_16 : 1_* ω7*c), C_12 : 0_ 3-OH, Summed Feature 8 (C_18 : 1_* ω7*c and/or C_18 : 1_* ω6*c), C_17 : 1_* ω8*c, C_17 : 0_ and C_12 : 0_. The major respiratory quinone was Q8 (97.8%). Polar lipids included aminophospholipid, phosphatidylethanolamine, phosphatidylglycerol, phospholipid and an unidentified lipid. The DNA G+C content of strain RL-SH01^T^ was 58.50 mol%. Based on morphological, physiological, chemotaxonomic and genomic evidence, strain RL-SH01^T^ represents a novel species of the genus *Gallaecimonas*, for which the name *Gallaecimonas zhanjiangensis* sp. nov. is proposed. The type strain is RL-SH01^T^ (=MCCC 1K10613^T^=KCTC 18644^T^). In general, the discovery of this new species enriches the germplasm resources of the genus *Gallaecimonas*, and broadens our understanding of its phylogenetic diversity and ecological distribution.

## Data Availability

The original contributions presented in this study are included in the article/supplementary material. Further inquiries can be directed to the corresponding author.

## Introduction

Mangrove ecosystems are typical wetland ecosystems distributed at the junction of land and sea in tropical and subtropical regions [1]. Due to the long-term dual effects of land and sea, mangrove sediments are characterized by salinization, acidity and water accumulation [2, 3]. When terrigenous pollutants enter mangroves via rivers, they are readily intercepted and continuously accumulate in sediments [4, 5]. Meanwhile, marine pollutants can also be transported into mangrove ecosystems via tidal action [6]. Subject to the combined pressures of terrestrial and marine pollution, pollutant-degrading bacteria gradually become enriched in mangrove sediments [7, 8]. Therefore, isolating and identifying these micro-organisms is of great significance for the understanding of the fate of pollutants in mangrove ecosystems and exploring microbial remediation of environmental pollution. To date, a large number of biologically active bacteria have been isolated from mangrove sediments for applications in biodegradation and agriculture [9, 10].

The genus *Gallaecimonas* belongs to the class Gammaproteobacteria. As of 6 June 2026, it includes four validly published names and one invalidly published name (https://lpsn.dsmz.de/genus/Gallaecimonas, accessed on 6 June 2026). Originally proposed by Rodríguez-Blanco *et al*., the type strain *Gallaecimonas pentaromativorans* CEE_131^T^ was isolated from sediment of the Corcubión Ría in Cee, A Coruña, Spain [11]. The remaining three validly published species are *Gallaecimonas xiamenensis* 3-C-1^T^, *Gallaecimonas mangrovi* HK-28^T^ and *Gallaecimonas kandeliae* Q10^T^, isolated from surface seawater (Xiamen, China), mangrove sediments (Hainan, China) and *Kandelia obovata* mangrove sediments (Shenzhen, China), respectively [12–14]. These findings indicate that the genus *Gallaecimonas* is primarily derived from marine ecosystems.

In this study, a bacterial strain, RL-SH01^T^, was isolated from mangrove sediments on Techeng Island of Zhanjiang City, China. Phylogenetic analysis based on the 16S rRNA gene sequences showed that strain RL-SH01^T^ belonged to the genus *Gallaecimonas*. It was characterized using polyphasic taxonomic methods (including morphology, physiology, biochemistry and chemotaxonomy) and is proposed as a new species of the genus *Gallaecimonas*, named *Gallaecimonas zhanjiangensis* sp. nov.

## Methods

### Sample collection and isolation of strain

Mangrove sediment samples used in this study were collected from Techeng Island of Zhanjiang City, China (21° 9′ 20″ N 110° 26′ 24″ E). During sampling, the overlying sediment was removed, sediment samples at 5–10 cm below the surface were collected, and large root residues and gravels were removed. Approximately 1 g of sediment was added to a liquid minimal salt medium (MSM) containing poly(butylene adipate-*co*-terephthalate) (PBAT) at 50 g l^−1^ as the sole carbon source and incubated at 30 °C and 180 r.p.m. for 7 days. Subsequently, the bacterial culture was streaked onto solid MSM containing PBAT (50 g l^−1^) and incubated at 30 °C for 7 days. A single colony capable of forming hydrolysis halos was selected and purified (Fig. S1, available in the online Supplementary Material). The isolated strain was routinely incubated in Luria–Bertani (LB) medium and stored in 30% (*v/v*) glycerol at −80 °C.

LB medium: NaCl 10 g l^−1^, tryptone 10 g l^−1^ and yeast extract 5 g l^−1^. MSM: NaCl 10 g l^−1^, (NH_4_)_2_SO_4_ 2 g l^−1^, MgSO_4_·7H_2_O 0.2 g l^−1^, Na_2_HPO_4_·12H_2_O 1.5 g l^−1^, KH_2_PO_4_ 1.5 g l^−1^, KNO_3_ 1 g l^−1^ and CaCl_2_ 0.01 g l^−1^.

The solid media of LB and MSM were prepared by supplementing agar with a final concentration of 15 g l^−1^. The pH of media was adjusted to 7.0±0.2, sterilized by autoclaving (121 °C, 30 min) and stored for subsequent use.

### 16S rRNA gene sequencing and phylogenetic analysis

The universal primers 27F (5′-AGAGTTTGATCCTGGCTCAG-3′) and 1492R (5′-TACCTTGTTACGACTT-3′) were used to amplify the 16S rRNA gene sequence by PCR. The PCR product was inserted into the pMD19-T vector (TaKaRa) and transformed into *E. coli* DH5α. Positive clones were screened, and recombinant plasmids were extracted. The recombinant plasmid was sent to GENEWIZ Biological Co., Ltd. (Guangzhou, China) for sequencing. Using the 16S Identification server of EzBioCloud (https://www.ezbiocloud.net/), we compared the almost-complete 16S rRNA gene sequences with similar sequences. The 16S rRNA gene sequences of each type strain were obtained from the National Center for Biotechnology Information (NCBI) database (https://www.ncbi.nlm.nih.gov/). Phylogenetic analysis of 16S rRNA gene sequences was performed using mega11 software. Sequence alignment was carried out using muscle. Genetic distances for the neighbour-joining (NJ) method were calculated based on the Maximum Composite Likelihood model, including both transitions and transversions. The bootstrap method with 1,000 replicates was used to evaluate the stability of the tree topology. Gap and missing bases in the alignment were treated by partial deletion, with a site coverage threshold of 50% [15]. To verify the robustness of the tree topology, the maximum-likelihood (ML) [16] and minimum-evolution (ME) [17] methods were also used for supplementary analyses.

### Morphological, physiological and biochemical characterization

The bacterial culture was streaked onto an LB plate and incubated at 30 °C for 12 h in the dark to observe colony morphology. Cell morphology was observed using a transmission electron microscope (TEM; Hitachi HT-7800, Japan). Reference type strains *G. xiamenensis* 3-C-1^T^ (MCCC 1A01354^T^), *G. mangrovi* HK-28^T^ (MCCC 1K03441^T^) and *G. kandeliae* Q10^T^ (MCCC 1K08421^T^) were purchased from the Marine Culture Collection of China (MCCC, China). *G. pentaromativorans* CEE_131^T^ (DSM 21945^T^) was purchased from the German Collection of Microorganisms and Cell Cultures GmbH (DSMZ, Germany). All strains were cultured under the same conditions. According to the single-variable principle, the isolated strain and the reference type strains were inoculated in LB medium at different temperatures (10–50 °C, at 5 °C intervals), initial pH values (4.0–11.0, at 0.5 pH-unit intervals) and NaCl concentrations (0–10%, *w*/*v*, at 0.5% intervals). After incubation for 12 h, the culture was sampled to measure the OD at 600 nm (OD_600_). Optimal growth conditions were defined as those resulting in an OD_600_ >2. All biological experiments were performed in triplicate. Gram staining, indole production, methyl red test, starch hydrolysis and gelatin hydrolysis were performed on the strains according to the Handbook for Systematic Identification of Common Bacteria [18]. A fresh bacterial suspension (OD_600_=1.0) was inoculated by stabbing into the centre of semi-solid LB medium containing 0.3% agar and then incubated at 30 °C for 24 h. Motility was evaluated by observing the turbid halo diffusing from the inoculation stab line. Catalase activity was determined by observing whether the strain produced bubbles in 3% (*v/v*) H_2_O_2_ solution. Oxidase activity was determined by the oxidation reaction of 1% (*w/v*) *p*-aminodimethylaniline oxalate. Biolog GEN III microplate was used to determine the ability of the isolated strain and the four reference type strains to utilize individual carbon sources.

### Chemotaxonomic characterization

The strain RL-SH01^T^ and four reference strains were cultured in LB liquid medium at 30 °C for 12 h to the logarithmic growth phase. Subsequently, the cells were collected by centrifugation for 10 min (4 °C, 8,000 ***g***), and the collected cells were freeze-dried for subsequent chemotaxonomic analysis. All subsequent experiments were carried out under the same conditions for the chemotaxonomic characterization of the isolated strain, *G. pentaromativorans* CEE_131^T^, *G. xiamenensis* 3-C-1^T^, *G. mangrovi* HK-28^T^ and *G. kandeliae* Q10^T^. Respiratory quinones were extracted from 100 mg of freeze-dried cells by adding 40 ml of chloroform-methanol (2 : 1, *v/v*), separated by TLC and identified by HPLC (Waters, USA) [19]. Fatty acid analysis was performed according to the standard procedure of the Sherlock Microbial Identification System (MIDI, version 6.0) and identified using the TSBA 6.0 database. Polar lipids were extracted using a chloroform/methanol system according to Kates’ method and analysed by one- and two-dimensional TLC [20]. Merck silica gel 60 F254 aluminium-backed thin-layer plates were used for TLC analysis. Total lipids were visualized using 10% ethanolic molybdophosphoric acid solution (*v/v*), glycolipids (GLs) with 1-naphthol sulphuric acid ethanol, aminolipids with ninhydrin and phospholipids with molybdenum blue reagent [19].

### Whole-genome analysis

The whole-genome sequencing of the isolated strain was done by Beijing Biomarker Technologies Co., Ltd. (Beijing, China). The experimental procedure was performed according to the standard operating protocol provided by Pacific Biosciences of California, Inc. (PacBio; California, USA). BUSCO (v5.8.2) and CheckM (v1.1.3) were used to evaluate genome assembly completeness and quality. The whole-genome assembly results were deposited to GenBase (https://ngdc.cncb.ac.cn/) to extract the 16S rRNA gene sequence of the strain. Subsequently, the extracted sequence was compared with the sequence obtained by 16S rRNA amplification and sequencing using the NCBI Nucleotide blast tool (https://blast.ncbi.nlm.nih.gov/Blast.cgi). To compare genome-relatedness, whole-genome sequences of the related type strains were obtained from the NCBI database (https://www.ncbi.nlm.nih.gov/). The G+C content of the strain was calculated based on the genomic sequence. Phylogenomic analysis was performed using the Genome Taxonomy Database Toolkit (GTDB-Tk v2.7.2) with the Genome Taxonomy Database (GTDB) release R232. The classify_wf workflow was run using default parameters [21]. The pipeline first identified a set of 120 bacterial single-copy marker genes (Bac120), aligned them with HMMER and concatenated the aligned sequences into a multi-sequence alignment matrix. This concatenated matrix was then used to place the query genome into the GTDB reference tree. In addition to the genome of strain RL-SH01^T^, the genome sequences of four reference type strains of the genus *Gallaecimonas* and one outgroup strain were included in the analysis. The resulting phylogenetic tree was rooted with *Gordonia hongkongensis* HKU50^T^ as the outgroup and visualized using FigTree v1.4.4. The average nucleotide identity (ANI) between the isolate and the reference type strains was calculated using the online ANI calculator of CJ Bioscience (https://www.ezbiocloud.net/tools/ani). Digital DNA–DNA hybridization (dDDH) values were calculated via the Type Strain Genome Server (TYGS, https://tygs.dsmz.de/).

### Accession numbers of nucleotide sequences

The BioProject and BioSample accession numbers for the whole-genome sequencing of strain RL-SH01^T^ are PRJNA1464122 and SAMN59683479, respectively. The complete genome assembly has been deposited under the accession number CM172603. Additionally, the 16S rRNA gene sequence of this strain is available under the accession number PZ387708.

## Results and discussion

### Phylogenetic analysis and genomic characteristics

The 16S rRNA sequence obtained by amplicon sequencing and the 16S rRNA sequence extracted from the whole-genome sequence were 1,528 and 1,533 bp in length, respectively. The two sequences shared 99.93% similarity, indicating that the obtained sequences were highly reliable and could be used for subsequent analyses. The results of 16S rRNA gene sequence alignment showed that the isolated strain RL-SH01^T^ (GenBank accession no. PZ387708) had the highest similarity (99.11%) with *G. pentaromativorans* CEE_131^T^ (GenBank accession no. FM955224). The similarities with *G. xiamenensis* 3-C-1^T^ (GenBank accession no. JQ666155), *G. kandeliae* Q10^T^ (GenBank accession no. OQ511015) and *G. mangrovi* HK-28^T^ (GenBank accession no. MF800856) were 97.26%, 96.98% and 96.84%, respectively. The NJ phylogenetic tree showed that strain RL-SH01^T^ formed an independent branch on the phylogenetic tree from other species of the genus *Gallaecimonas* (Fig. 1). The topologies of the ML (Fig. S2) and ME trees (Fig. S3) are similar to that of the NJ tree.

**Fig. 1.**
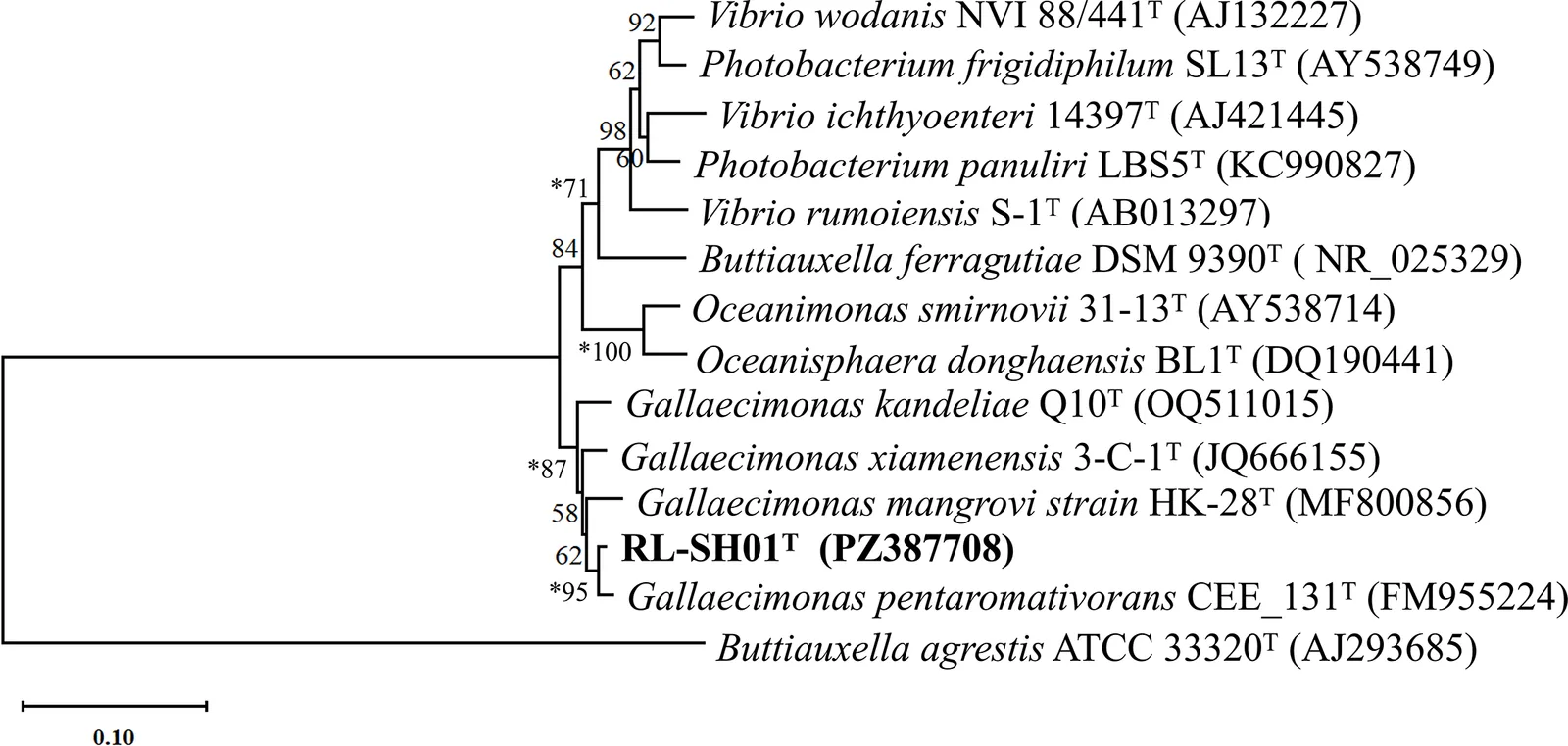
NJ phylogenetic tree based on 16S rRNA gene sequences. Bootstrap values are based on 1,000 replicates. The scale bar represents 0.1 substitutions per nucleotide position. The node marked by the asterisk (*) indicates that the branch has the same topological structure in the ML method and the ME method phylogenetic trees, and the bootstrap value is ≥70%. T indicates the type strain.

The genome sequencing results showed that the genome coverage was 100.0%, and the average sequencing depth was 80.42×. BUSCO was used to evaluate the completeness of the genome assembly, and the results showed that a total of 115 complete BUSCO genes were detected, with a complete gene ratio of 99.1%. The genome quality was further evaluated using CheckM, and the results showed that the genome completeness was 99.82%, the contamination was 0.0%, and the strain heterogeneity was 0.0%. These results indicate that the assembled genome has very high integrity and purity, fully meeting the requirements for subsequent phylogenomic analysis. The complete genome of strain RL-SH01^T^ is a circular chromosome of 4,322,905 bp with a G+C content of 58.50 mol%. Phylogenetic analysis based on 120 bacterial single-copy marker genes showed that strain RL-SH01^T^ and *G. pentaromativorans* CEE_131^T^ formed the same phylogenetic branch within the genus (Fig. S4). The ANI value between RL-SH01^T^ and *G. pentaromativorans* CEE_131^T^ was 95.1% (with an alignment coverage of 0.887), which fell within the critical range for prokaryotic species delineation (95%–96%). The ANI values for the other three *Gallaecimonas* strains were below the threshold (Table 1). To further clarify its taxonomic status, we calculated the dDDH value based on genomic data. The results showed that the dDDH value between strain RL-SH01^T^ and *G. pentaromativorans* CEE_131^T^ was 62.7%, which is below the species delineation threshold (70%). Based on the phylogenomic, ANI and dDDH analyses, strain RL-SH01^T^ formed an independent branch within the genus *Gallaecimonas*, with genomic differences from related species reaching the species delineation threshold. Therefore, the morphological, physiological and biochemical characteristics of the strain were further systematically characterized to verify its novel species status.

**Table 1.** 16S rRNA gene sequence similarity, ANI and dDDH values between strain RL-SH01T and *Gallaecimonas* strains

Strains	NCBI BioSample accession no.	Total length (bp)	G+C (mol%)	16S rRNA similarity (%)	ANI (%)	dDDH (%)
RL-SH01^T^	SAMN59683479	4,322,905	58.50	-	-	-
DSM 21945^T^	SAMN10363320	4,190,752	58.45	99.11	95.10	62.7
MCCC 1A01354^T^	SAMN02471169	4,062,282	60.58	97.26	80.52	22.8
MCCC 1K03441^T^	SAMN09742667	4,071,977	52.72	96.98	77.40	21.0
MCCC 1K08421^T^	SAMN33330377	3,836,841	62.57	96.84	78.22	29.4

DSM 21945T, *G. pentaromativorans* CEE_131T; MCCC 1A01354T, *G. xiamenensis* 3-C-1T; MCCC 1K03441T, *G. mangrovi* HK-28T; MCCC 1K08421T, *G. kandeliae* Q10T.

### Morphological, physiological and biochemical characteristics

The colonies of strain RL-SH01^T^ were light yellow, round and convex, with a diameter of 1–1.5 mm after incubation on LB medium at 30 °C in the dark for 12 h. TEM imaging indicated that the cells of strain RL-SH01^T^ were rod-shaped and flagellated, with a size of 0.5–0.6×1.2–1.8 µm (Fig. S5). Consistent with this flagellar morphology, the semi-solid medium stab culture assay confirmed that the strain was motile. Gram staining showed that strain RL-SH01^T^ is Gram-negative. Growth characteristic tests indicated that strain RL-SH01^T^ could grow at temperatures of 15–35 °C, salinities of 0–8.0% (*w/v*) and pH values of 4.0–10.0. The optimal growth conditions were 25–35 °C, 0.5–5.0% NaCl (*w/v*) and pH 5.0–9.0. In physiological and biochemical tests, oxidase, catalase, starch hydrolysis, gelatin hydrolysis and methyl red tests were positive, while the indole production test was negative. In the Biolog GEN III microplate test, the carbon source utilization results were as follows: substrates dextrin, d-maltose, d-trehalose, *N*-acetyl-d-glucosamine, *α*-d-glucose, d-glucose-6-PO_4_, d-fructose-6-PO_4_, gelatin, glycyl-l-proline, l-alanine, pectin, d-galacturonic acid, l-galactonic acid lactone, glucuronamide, methyl pyruvate, l-lactic acid, citric acid, *α*-keto-glutaric acid, l-malic acid, bromo-succinic acid, Tween 40, *α*-hydroxy-butyric acid, *β*-hydroxy-d,l-butyric acid, *α*-keto-butyric acid, acetoacetic acid, formic acid, acetic acid and propionic acid were positive; substrates *N*-acetyl-*β*-d-mannosamine, d-fructose, d-fucose, l-fucose, l-rhamnose, inosine, d-serine, l-glutamic acid, l-histidine, l-serine, d-gluconic acid, d-glucuronic acid, mucic acid and d-lactic acid methyl ester were weakly positive; and the remaining substrates were negative. The chemical sensitivity results were as follows: compounds 1% sodium lactate, fusidic acid, d-serine, rifamycin SV, lincomycin, guanidine HCl, Niaproof 4, vancomycin, tetrazolium violet, tetrazolium blue, nalidixic acid, lithium chloride, potassium tellurite, aztreonam and sodium butyrate were positive; compound sodium bromate was weakly positive; and the remaining test compounds were negative. The morphological, physiological and biochemical characteristics of strain RL-SH01^T^ and the reference type strains are summarized in Table 2.

**Table 2.** Morphological, physiological and chemotaxonomic characteristics of strain RL-SH01^T^ and reference type strains

Characteristic	RL-SH01^T^	DSM 21945^T^	MCCC 1A01354^T^	MCCC 1K03441^T^	MCCC 1K08421^T^
Cell size (μm)	0.5–0.6×1.2–1.8	0.3–0.5×1.4–2.9^a^	0.4–0.7×2.2–4.1^b^	0.7–0.9×1.1–3.8^c^	0.3–0.5×1.2–2.0^d^
Flagella	+	+^a^	+^b^	-^c^	-^d^
Temperature range (optimum) (°C)	15–35 (25–35)	15–40 (25–35)	15–40 (25–35)	15–40 (30–35)	15–50 (25–35)
pH range (optimum)	4.0–10.0 (5.0–9.0)	5.0–10.0 (5.5–8.0)	5.0–10.5 (5.5–10)	5.0–8.0 (6.0–7.0)	5.0–8.0 (6.0–7.5)
NaCl range (optimum) (%, *w/v*)	0–8.0 (0.5–5.0)	0–7.0 (1.0–5.0)	0–7.0 (0–5.5)	0.5–7.0 (1.5–3)	0–8.0 (0.5–3.0)
Oxidase	+	−	+	−	+
Hydrolysis of starch	+	+	+	−	+
Gelatin hydrolysis	+	+	+	−	−
Utilization of substrates in Biolog GEN III carbon source utilization tests					
d-Trehalose	+	W	−	−	−
d-Serine	+	−	−	−	W
Glucuronamide, pectin	+	−	−	−	−
l-Lactic acid	+	+	−	+	−
Citric acid	+	+	+	−	W
*α*-Hydroxy-butyric acid	+	+	+	+	−
Acetoacetic acid	+	+	−	+	+
Formic acid	+	+	−	−	−
*N*-Acetyl-*β*-d-mannosamine, l-histidine	W	−	+	−	+
d-Fructose, l-fucose	W	+	W	−	+
d-Fucose	W	+	+	+	+
l-Rhamnose	W	W	W	W	+
Inosine	W	+	+	+	−
l-Glutamic acid	W	+	W	+	+
l-Serine	W	+	+	−	+
d-Gluconic acid, mucic acid, d-lactic acid methyl ester	W	−	−	−	W
Turanose	−	W	−	−	+
*N*-Acetyl-d-galactosamine, l-arginine	−	−	−	−	+
Gentiobiose	−	−	W	−	W
d-Cellobiose	−	+	−	+	W
Glycerol	−	+	−	−	W
l-Aspartic acid	−	−	+	+	−
Biolog GEN III chemical sensitivity tests					
Fusidic acid	+	+	+	−	W
Tetrazolium violet	+	+	+	−	+
Minocycline	−	+	+	W	−
Guanidine HCl, Niaproof 4	+	+	+	−	−
Nalidixic acid	+	−	+	+	+
Respiratory quinone	Q7, Q8	Q7, Q8, Q9^c^	Q7, Q8, Q9^c^	Q7, Q8^c^	Q8^d^
Polar lipids	APL, PE, PG, PL, L	DPG, PG, PE, APL1, APL2, PL1, PL2, PL3, GL1, GPL^c^	DPG, PG, PE, APL1, APL2, PL1, PL2, PL3, GL1, GPL^c^	DPG, PG, PE, APL1, APL2, PL1, PL2, PL3, PL4, GL1, GL2, GPL, AL, L^c^	DPG, PG, PE, APL, PL, GL, GPL, AL^d^

DSM 21945T, *G. pentaromativorans* CEE_131T; MCCC 1A01354T, *G. xiamenensis* 3-C-1T; MCCC 1K03441T, *G. mangrovi* HK-28T; MCCC 1K08421T, *G. kandeliae* Q10T.

The data marked a, b, c and d in the table are cited from references [11–13] and [14], respectively; the remaining data are from this study.

+, Positive; −, negative; W, weakly positive; wells showing colour weaker than that of the positive control but still visible on day 2 are recorded as weakly positive. Negative wells at day 2 that turn dark by day 7 after prolonged incubation are reclassified as weakly positive.

AL, aminolipid; APL, aminophospholipid; DPG, diphosphatidylglycerol; GL, glycolipid; GPL, glycophospholipid; L, unknown polar lipid; PE, phosphatidylethanolamine; PG, phosphatidylglycerol; PL, phospholipid.

### Chemotaxonomic characterization

The major fatty acids of strain RL-SH01^T^ were C_16 : 0_ (22.0%), Summed Feature 3 (C_16 : 1_* ω6*c and/or C_16 : 1_* ω7*c; 13.4%), C_12 : 0_ 3-OH (10.5%), Summed Feature 8 (C_18 : 1_* ω7*c and/or C_18 : 1_* ω6*c; 9.0%), C_17 : 1_* ω8*c (8.4%), C_17 : 0_ (8.1%) and C_12 : 0_ (5.4%). The complete fatty acid profile is summarized in Table 3. The major respiratory quinone of strain RL-SH01^T^ was Q8, accounting for 97.8% of the total. A small amount of Q7 (2.2%) was also detected, whereas Q9 was not detected. The polar lipids of strain RL-SH01^T^ included aminophospholipid (APL), phosphatidylethanolamine (PE), phosphatidylglycerol (PG), phospholipid (PL) and an unknown polar lipid (L). The polar lipid profile of strain RL-SH01^T^ was similar to those of other members of the genus *Gallaecimonas*, but GLs were not detected (Fig. S6).

**Table 3.** Cellular fatty acid composition of strain RL-SH01^T^ and reference type strains

Fatty acid	RL-SH01^T^	DSM 21945^T^	MCCC 1A01354^T^	MCCC 1K03441^T^	MCCC 1K08421^T^
Saturated					
C_10 : 0_	tr	–	1.3	–	–
C_12 : 0_	5.4	3.3	3.2	3.6	2.2
C_13 : 0_	1.8	–	–	–	1.3
C_14 : 0_	2.3	3.2	3.8	2.5	tr
C_16 : 0_	22.0	27.5	24.8	26.2	15.8
C_17 : 0_	8.1	1.5	4.0	4.6	5.9
C_18 : 0_	tr	1.6	1.1	1.8	2.1
Branched					
iso-C_14 : 0_	1.9	3.0	2.7	tr	8.0
anteiso-C_15 : 0_	tr	1.2	tr	tr	2.2
iso-C_16 : 0_	3.3	4.9	3.8	1.3	11.1
iso-C_17 : 0_	tr	1.3	tr	tr	tr
iso-C_18 : 0_	tr	tr	1.0	1.1	1.5
Hydroxy					
C_11 : 0_ 3-OH	2.8	tr	tr	tr	tr
C_12 : 0_ 3-OH	10.5	7.2	7.5	7.3	2.8
iso-C_12 : 0_ 3-OH	1.0	1.1	1.3	tr	1.7
Unsaturated					
C_15 : 1_* ω8*c	1.3	tr	1.3	tr	2.6
C_17 : 1_* ω6*c	–	–	–	–	tr
C_17 : 1_* ω8*c	8.4	2.5	4.4	4.6	15.0
C_17 : 0_ cyclo	2.0	2.2	–	tr	–
C_18 : 1_* ω9*c	tr	–	–	1.5	tr
C_19 : 0_ cyclo *ω*8c	tr	tr	–	2.2	–
Summed Feature 3	13.4	20.9	17.2	17.3	12.2
Summed Feature 8	9.0	12.7	15.7	19.7	8.1

DSM 21945T, *G. pentaromativorans* CEE_131T; MCCC 1A01354T, *G. xiamenensis* 3-C-1T; MCCC 1K03441T, *G. mangrovi* HK-28T; MCCC 1K08421T, *G. kandeliae* Q10T.

Summed Feature 3 contains C_16 : 1_* ω6*c and/or C_16 : 1_* ω7*c; Summed Feature 8 contains C_18 : 1_* ω7*c and/or C_18 : 1_* ω6*c.

–, not detected; tr, traces (< 1.0%).

## Description of *Gallaecimonas zhanjiangensis* sp. nov.

*Gallaecimonas zhanjiangensis* (zhan.ji.ang.en′sis. N.L. fem. adj. *zhanjiangensis*, of Zhanjiang, a city in Guangdong, China, close to where the type strain was isolated).

Cells of strain RL-SH01^T^ are Gram-negative, rod-shaped and flagellated, with a size of ~0.5–0.6×1.2–1.8 µm. After incubation on LB medium at 30 °C for 12 h, convex, round, pale yellow colonies with a diameter of ~1–1.5 mm were formed. The growth temperature range of the strain was 15–35 °C (optimum, 25–35 °C). It could grow at salinities of 0–8.0% (*w/v*), with an optimum of 0.5–5.0% (*w/v*). The growth pH range was 4.0–10.0 (optimum pH 5.0–9.0). Oxidase, catalase, starch hydrolysis, gelatin hydrolysis and methyl red tests were positive, whereas the indole production test was negative. The major respiratory quinone was Q8, with a trace amount of Q7. The major fatty acids (>5%) were C_16 : 0_, Summed Feature 3, C_12 : 0_ 3-OH, Summed Feature 8, C_17 : 1_* ω8*c, C_17 : 0_ and C_12 : 0_. The polar lipids included APL, PE, PG, phospholipid and an unidentified lipid. In the Biolog GEN III microplate, strain RL-SH01^T^ was able to utilize dextrin, d-maltose, d-trehalose, *N*-acetyl-d-glucosamine, *N*-acetyl-*β*-d-mannosamine, *α*-d-glucose, d-fructose, d-fucose, l-fucose, l-rhamnose, inosine, d-glucose-6-PO_4_, d-fructose-6-PO_4_, d-serine, glycyl-l-proline, l-alanine, l-glutamic acid, l-histidine, l-serine, pectin, d-galacturonic acid, l-galactonic acid lactone, d-gluconic acid, d-glucuronic acid, glucuronamide, mucic acid, methyl pyruvate, d-lactic acid methyl ester, l-lactic acid, citric acid, *α*-keto-glutaric acid, l-malic acid, bromo-succinic acid, Tween 40, *α*-hydroxy-butyric acid, *β*-hydroxy-d,l-butyric acid, *α*-keto-butyric acid, acetoacetic acid, formic acid, acetic acid and propionic acid as the sole carbon source but could not assimilate the other tested substrates.

The type strain, RL-SH01^T^ (=MCCC 1K10613^T^=KCTC 18644^T^), was isolated from mangrove sediments of Techeng Island, Zhanjiang City, Guangdong Province, China. The genomic DNA G+C content of the type strain is 58.50 mol%. The 16S rRNA gene sequence (accession no. PZ387708) and the complete genome sequence (accession no. CM172603) were deposited in the NCBI GenBank database.

## Supplementary material

10.1099/ijsem.0.007353 Supplementary Material 1.

## Abbreviations

ANI: average nucleotide identity
APL: aminophospholipid
dDDH: digital DNA–DNA hybridization
GLs: glycolipids
GTDB: Genome Taxonomy Database
L: unknown polar lipid
MCCC: Marine Culture Collection of China
ME: minimum-evolution
ML: maximum-likelihood
MSM: minimal salt medium
NCBI: National Center for Biotechnology Information
NJ: neighbour-joining
OD_600_: OD at 600 nm
PBAT: poly(butylene adipate-co-terephthalate)
PE: phosphatidylethanolamine
PG: phosphatidylglycerol
PL: phospholipid
TEM: transmission electron microscope
