# Supplementary material for: Gallaecimonas zhanjiangensis sp. nov., a novel member of the phylum Pseudomonadota isolated from mangrove sediments
Source: Int J Syst Evol Microbiol. 2026 Nov 12;76(11):007353. doi: 10.1099/ijsem.0.007353 (PMC13760616; doi:10.1099/ijsem.0.007353)
Supplement: Supplementary file 1 [file ijsem-76-07353-s001.pdf]

Gallaecimonas zhanjiangensis sp. nov., a novel member of the phylum Pseudomonadota isolated from mangrove sediments

Min Shi[a], Yanyan Wang[a], Hanqiao Hu[a], Yang Jia[b], Baisuo Zhao[c], John L. Zhou[d], Lei Ren[a, *]

a College of Coastal Agricultural Sciences, Guangdong Ocean University, Zhanjiang 524088, China

b College of Life and Environmental Science, Wenzhou University, Wenzhou 325035, China

c Graduate School of Chinese Academy of Agricultural Sciences, Beijing 100081, China

d Faculty of Science and Engineering, University of Nottingham Ningbo China, Ningbo 315100, China

* Corresponding author:

Lei Ren, College of Coastal Agricultural Sciences, Guangdong Ocean University, Zhanjiang 524088, China, e-mail address: renlei@gdou.edu.cn

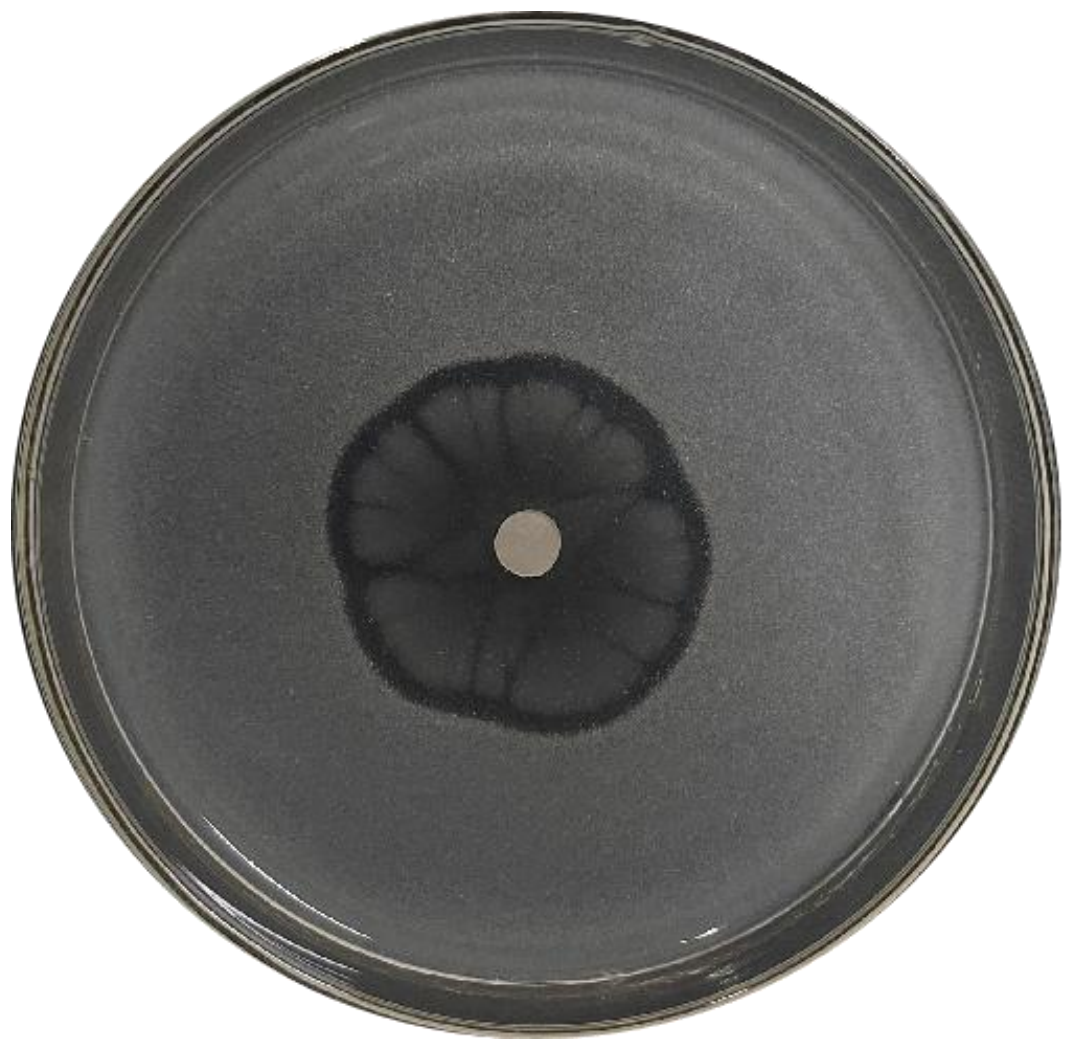

Figure S1 Hydrolysis halos produced by strain RL-SH01^T on mineral salt medium agar plates supplemented with PBAT as the sole carbon source after 7 days of incubation.

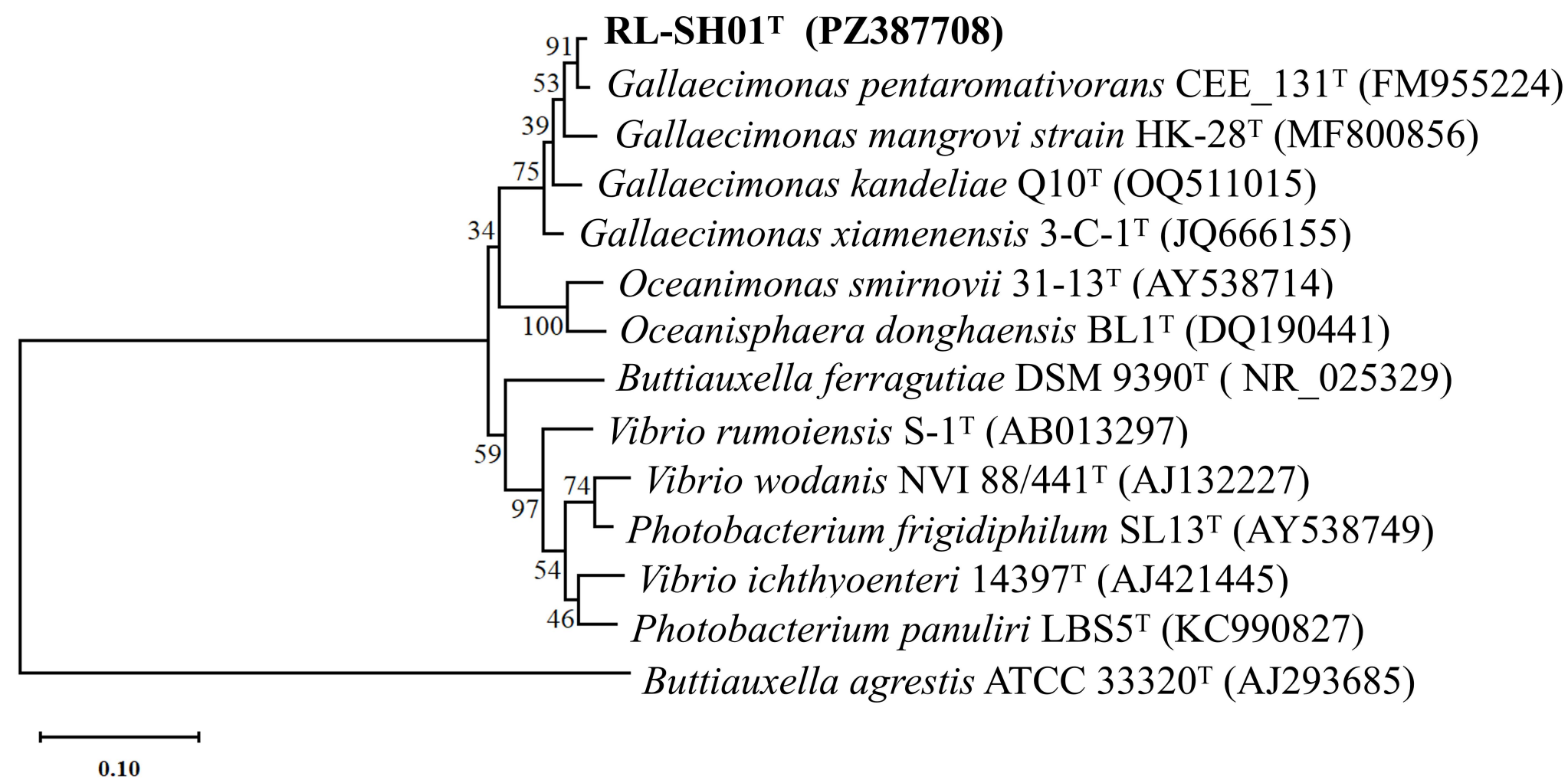

Figure S2 Maximum-likelihood phylogenetic tree based on 16S rRNA gene sequences. Bootstrap values are based on 1,000 replicates. The scale bar represents 0.1 substitutions per nucleotide position. T indicates the type strain.

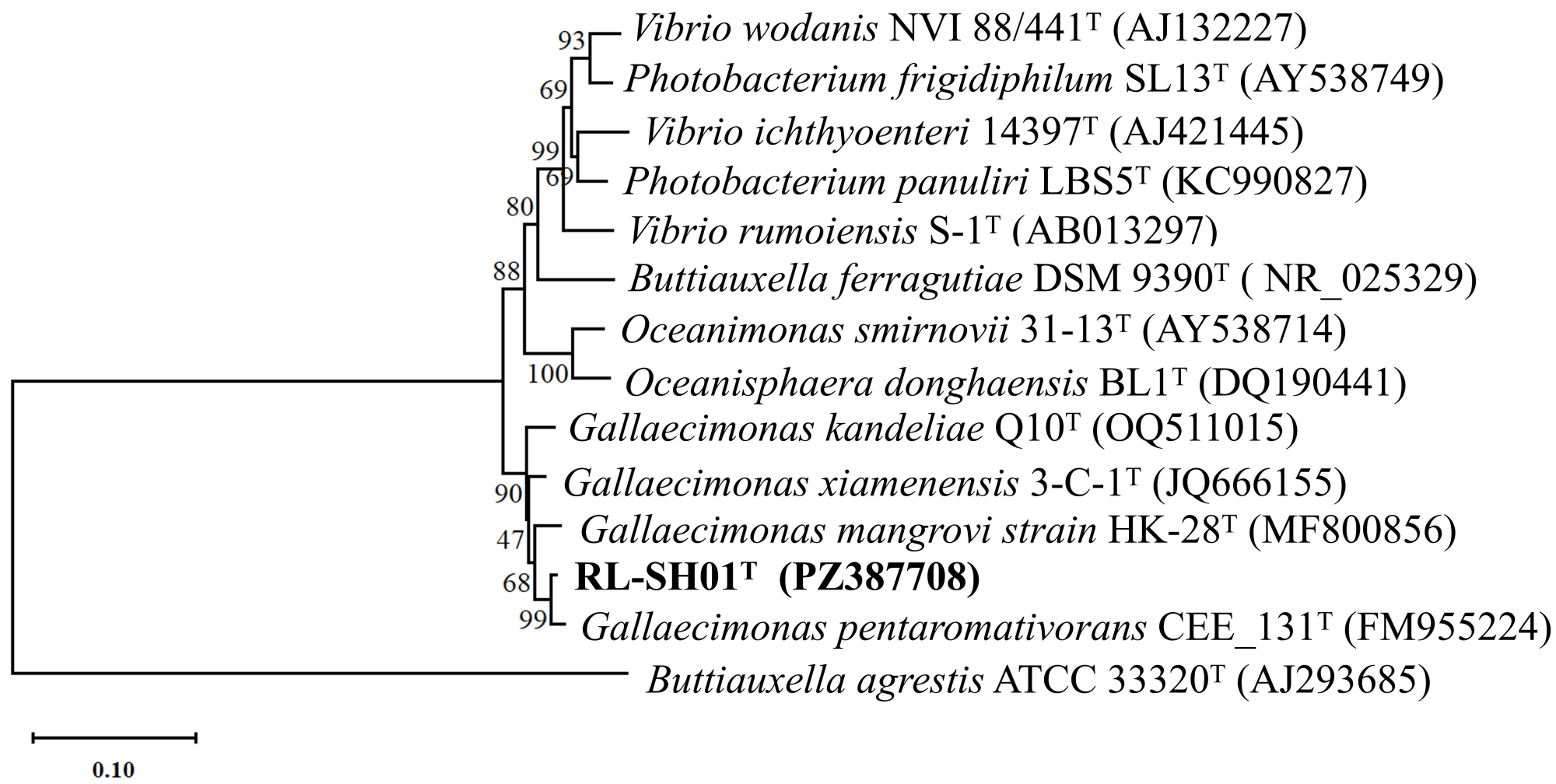

Figure S3 Maximum-Evolution phylogenetic tree based on 16S rRNA gene sequences. Bootstrap values are based on 1,000 replicates. The scale bar represents 0.1 substitutions per nucleotide position. T indicates the type strain.

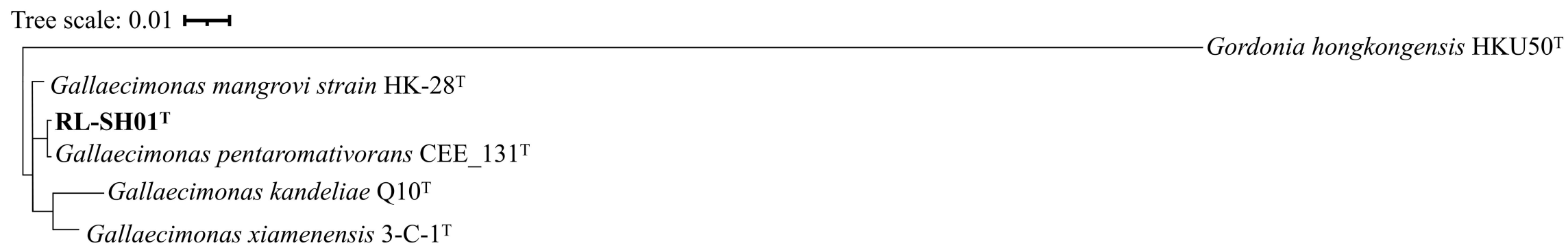

Figure S4 Phylogenomic tree of strain RL-SH01^{T} and related species of the genus *Gallaecimonas*, constructed using GTDB-Tk (v2.7.2; GTDB R232) based on the concatenated alignment of 120 bacterial single- copy marker genes. The scale bar represents 0.01 substitutions per site. T indicates the type strain.

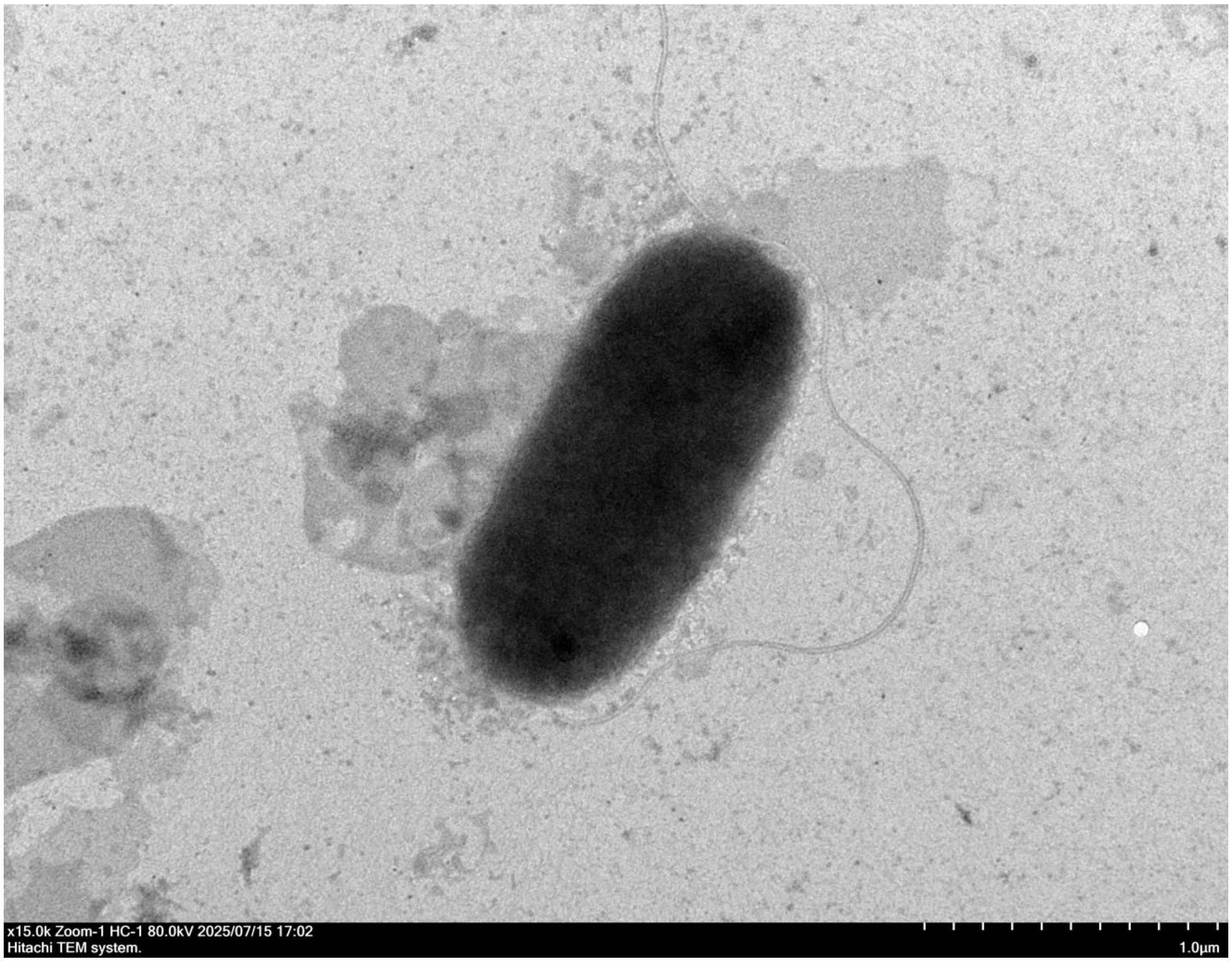

Figure S5 Transmission electron micrograph (TEM) of strain RL-SH01^T cultivated in LB medium at 30 °C for 24 h, revealing a single polar flagellum. Scale bar: 1 µm.

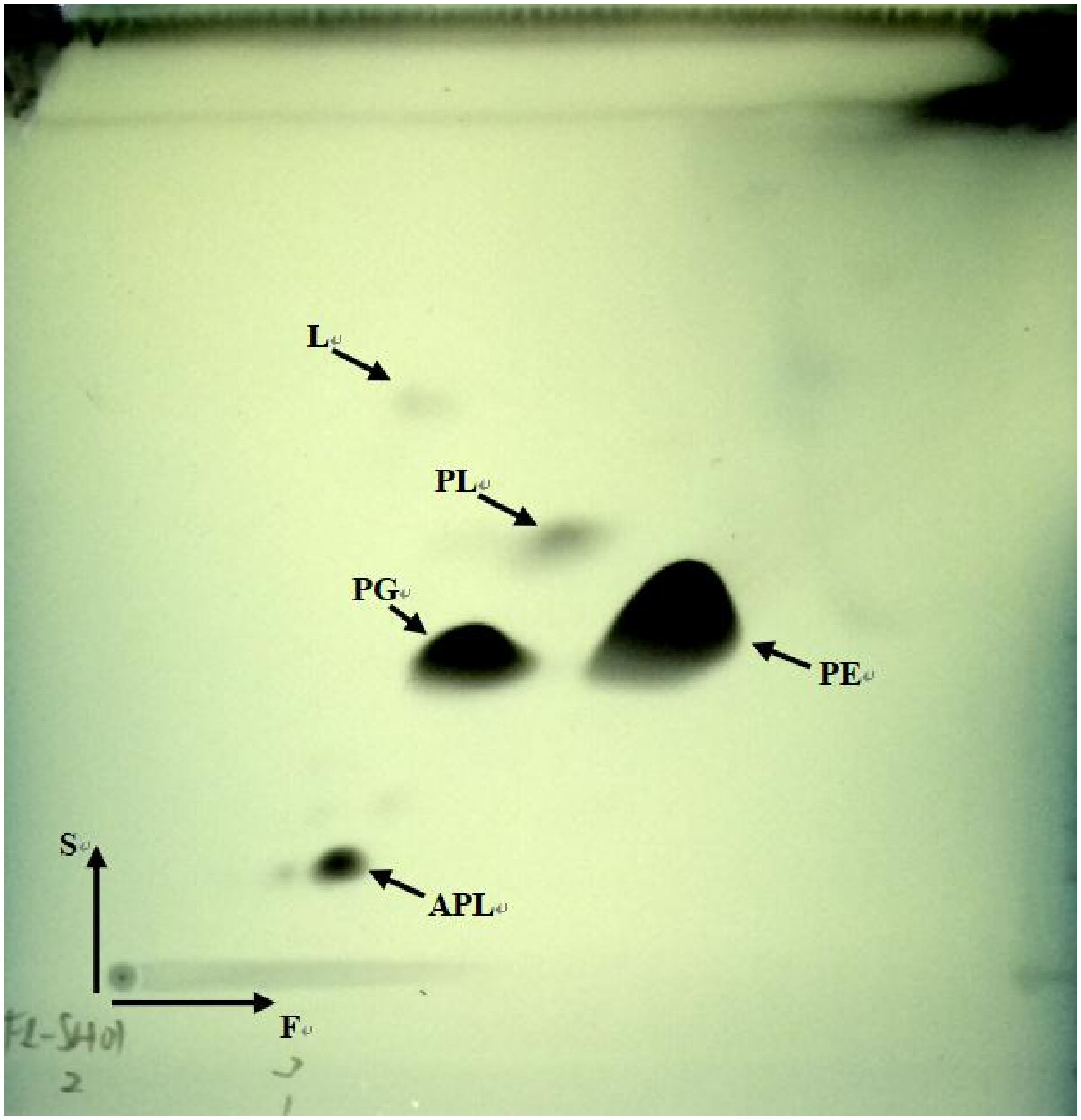

Figure S6 Polar lipid profile of strain RL-SH01. APL, aminophospholipid; L, unknown polar lipid; PE, phosphatidylethanolamine; PG, phosphatidylglycerol; PL, phospholipid.
